# A Chemokine Targets the Nucleus: Cxcl12-Gamma Isoform Localizes to the Nucleolus in Adult Mouse Heart

**DOI:** 10.1371/journal.pone.0007570

**Published:** 2009-10-27

**Authors:** Raul Torres, Juan C. Ramirez

**Affiliations:** Viral Vector Facility, Technical Unit of Gene Targeting, Fundacion CNIC (National Centre for Cardiovascular Research), Madrid, Spain; University of Hong Kong, Hong Kong

## Abstract

Chemokines are extracellular mediators of complex regulatory circuits involved principally in cell-to-cell communication. Most studies to date of the essential chemokine Cxcl12 (Sdf-1) have focused on the ubiquitously expressed secreted isoforms α and β. Here we show that, unlike these isoforms and all other known chemokines, the alternatively transcribed γ isoform is an intracellular protein that localizes to the nucleolus in differentiated mouse Cardiac tissue. Our results demonstrate that nucleolar transportation is encoded by a nucleolar-localization signal in the unique carboxy-terminal region of Sdf-1γ, and is competent both in vivo and in vitro. The molecular mechanism underlying these unusual chemokine properties involves cardiac-specific transcription of an mRNA containing a unique short-leader sequence lacking the signal peptide and translation from a non-canonical CUG codon. Our results provide an example of genome economy even for essential and highly conserved genes such as Cxcl12, and suggest that chemokines can exert tissue specific functions unrelated to cell-to-cell communication.

## Introduction

Chemoattractant cytokines, known as chemokines, are a class of small proteins that play key roles in intercellular signalling and cell migration throughout animal development and during adult life [1]. The chemokine Cxcl12, also known as stromal cell-derived factor 1 (Sdf-1) is a member of the CXC chemokine family, and is responsible for a variety of processes central to homeostasis and physiology [2], [3], [4] through binding to the seven transmembrane domain, G-protein coupled family of receptors (GPCR) Cxcr4 [5] and Cxcr7 [6]. Cxcr4 intracellular responses to Cxcl12/Sdf-1 involve signal transduction via PI3K, PLC/PKC, and MAPKp24/44 (ERK1/2), stimulating pathways associated with cell survival, whereas recent findings in zebrafish suggest that Cxcr7 functions primarily by sequestering Cxc12. CxcR7 heterodimerizes with CxcR4 and regulates Cxcl12-mediated G protein signaling [7]

Gene deletion of *Cxcl12*, *Cxcr4* or *Cxcr7* results in embryonic lethality from E18.5 and is associated with severe developmental defects affecting the central nervous system, heart and vasculature [8], [9], [10], [11]. Three isoforms of Cxcl12/Sdf-1, produced from alternatively spliced mRNA variants, have been identified in humans, rats and mice [12], [13], [14]. Sdf-1α and β were the first isoforms identified, and most available data on Sdf-1 were obtained with the α isoform. These isoforms are highly similar, distinguished by a difference in just four amino acids in the C-terminus and a larger 3′UTR in *Sdf-1β*. This similarity is reflected in the minor differences between their biological properties [12]. Both proteins are secreted via the canonical intracellular secretory pathway, mediated by a signal peptide sequence.

The γ isoform was identified more recently, together with other minor isoforms in humans (δ, ε, and φ) [13]. Preliminary data from humans show that SDF-1γ expression is mainly restricted to regions of adult brain and heart, with a similar pattern seen in rats [14], [15]. However, the biological significance of Sdf-1γ has been unclear, since it binds Cxcr4 with low affinity and displaces Cxcr4-bound Sdf-1α only at high concentrations [16]. Moreover, it is ineffective at driving signal transduction, as revealed by its low capacity to stimulate intracellular calcium mobilization, weak chemotactic activity and lack of effect on progenitor cell survival [17]. Contrasting with the sequence similarity of Sdf-1α and β, Sdf-1γ contains a unique thirty amino-acid sequence at its C terminus, making it almost twice as large as Sdf-1α and β (14 versus 8 kDa) [18]. Moreover, almost 60% of the residues in this C-terminal extension are basic (lysine and arginine). Enrichment in basic residues is a characteristic of protein domains involved in intermolecular interactions with DNA, lipids, sugars and other proteins, and in translocation across membranes during localization to subcellular compartments. The lysine and arginine residues in the Sdf-1γ C-terminal region are arrayed in at least four clusters resembling the canonical nuclear localization signals (NLS) of cellular and viral proteins [19], a feature not described for any other chemokine. Recent reports describe extracellular actions for two of these clusters as heparan-sulphate binding domains, with unexpectedly strong binding affinity for cellular glycosaminoglycans (GAGs) [16], [20]. However, all these experiments were conducted with in vitro synthesized recombinant Sdf-1γ, and activity derived from the expression from the endogenous locus has not been reported.

Here, we demonstrate that Sdf-1γ is a nuclear protein in the mouse heart, where it is expressed in a temporally coordinated pattern during development and at high levels postnatally and in adults. Our results show that the Sdf-1γ C-terminal region localizes the protein to the nucleus via canonical NLS motifs and to the nucleolus via a specific nucleolar localization signal (NoLS). The Sdf-1γ C-terminal extension moreover confers nuclear localization on heterologous cytoplasmic proteins. We have demonstrated that the *Sdf-1γ* mRNA in cardiac tissue has a short (25 nt) leader sequence which lacks a signal peptide sequence and the AUG initiation codon used to translate *Sdf-1α* and *β* mRNAs. Sdf-1γ is instead translated from the non-canonical codon CUG at position 169. These findings establish Sdf-1γ in a different class from all known chemokines, as a member of the nuclear proteome [21], and introduce the novel idea that chemokines can exert intracellular signalling functions not directly related to intercellular signalling.

## Results

### *Sdf-1γ* is predominantly expressed in the heart in mice

**Figure 1:**
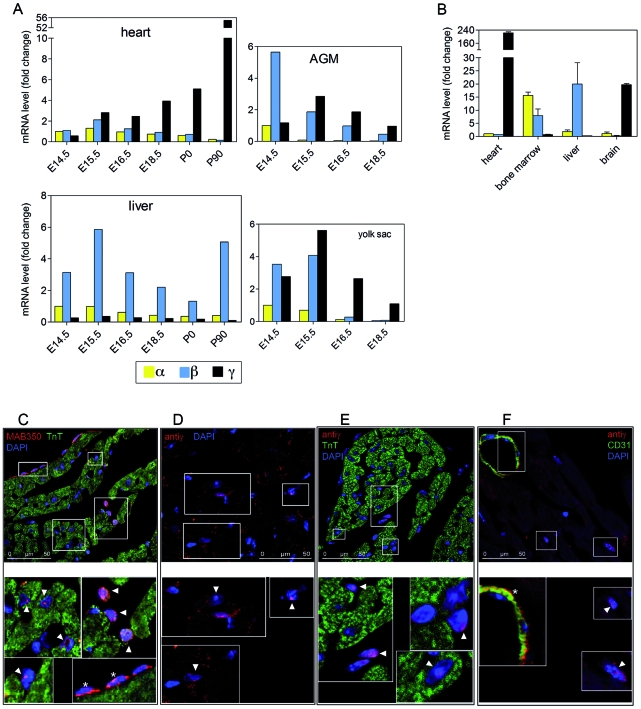
Isoform γ is the predominant Sdf-1 isoform expressed in the postnatal mouse heart. (A) mRNA expression of the main *Sdf-1* isoforms during late embryonic development and postnatally. Expression levels of *Sdf-1*α, *Sdf-1*β and *Sdf-1*γ were measured by quantitative real-time PCR on total RNA extracted from the indicated tissues at the indicated times. For each tissue, mRNA amounts were normalized to *Sdf-1*α expression at E14.5 ( = 1). AGM: aorta–gonad–mesonephros (B) Comparison of *Sdf-1* isoform mRNA expression in adult (P90) tissues (qRT-PCR). RNA amounts in each tissue were normalized to *Sdf-1*α expression ( = 1). For embryo samples, RNA was pooled from at least five littermates; for postnatal stages, samples from five age-matched individuals at P0 or P90 were pooled. Analysis at each stage was repeated three times, yielding similar results (n = 5). (C to F) Confocal immunofluorescence of cardiac expression of Sdf-1γ in adult mice (P90). Thin (5 µm) cryosections of three-month old mouse heart were stained for Sdf-1 (red) with anti-pan Sdf-1 antibody (MAB350; C) or specific anti Sdf-1γ antibody (anti-γ; D,E,F). Green staining shows immunofluorescence of troponin T alpha (C, E) or CD31 (F). Nuclei were stained with DAPI (blue). Lower panels show high-magnification images of the boxed areas in the upper panels. Nuclear staining with anti-Sdf-1 antibodies is indicated by arrowheads (non-endothelial cells) or asterisks (endothelial cells). Note the nucleolar staining with anti-Sdf-1 antibodies, seen as spots inside nuclei.

Published data on the human and murine expression patterns of *Sdf-1* family members are scarce, and do not provide systematic, quantified or sufficiently detailed information [13], [18], [20], [22]. We investigated the expression patterns of *Sdf-1*α, β and γ mRNAs during mouse development to gain insight into possible isoform specificity. For this, we designed primers to target exons specific to each alternately-spliced isoform (Fig. S1), and used these to probe total RNA by qRT-PCR. For embryos, RNA was extracted from liver, heart, aorta–gonad–mesonephros (AGM) and yolk sac at stages E14.5, E15.5, E16.5 and E18.5. For later stages, RNA was extracted from liver, heart, brain, and bone marrow of newborns (P0) and adults (3-months old). Expression of *Sdf-1*α did not vary significantly between organs or with developmental stage, whereas *Sdf-1*β and *Sdf-1*γ did (Fig. 1A). For comparisons, the expression of *Sdf-1*α at E14.5 within each tissue was set as the baseline. Expression of *Sdf-1*γ increased from E15.5, becoming the most abundant *Sdf-1* transcript at E18.5 in the developing heart, AGM and yolk sac. *Sdf1*β is the main isoform expressed in liver, with *Sdf-1*γ and α barely detectable at all times studied. Expression of *Sdf-1*γ mRNA increased throughout development, reaching a maximum at birth.

Adult mice (3 months) showed a marked tissue-specific expression of *Sdf-1* isoforms. *Sdf-1*β expression is prominent in hematopoietic tissues (bone marrow) and liver. In contrast, *Sdf-1*γ is abundant in brain and especially in heart: *Sdf-1*γ mRNA levels in these organs were respectively ∼27-fold and ∼260-fold above those of *Sdf-1*α (Fig. 1B). These findings support the idea that Sdf-1γ can be considered a predominantly cardiac version of the chemokine Cxcl12.

### Sdf-1γ localizes to the nucleus of cardiomyocytes

To examine protein expression of Sdf-1γ in heart by immunohistochemistry, a specific anti-Sdf-1γ antibody (hereafter called anti-γ) was raised in rabbit against a peptide sequence from the Sdf-1γ carboxy-terminal end (Fig. S2) and affinity purified before use. Antibody specificity and reactivity were analyzed by Western blot of extracts of HEK293T cells transfected with constructs pSdf1α-30–359 or pSdf1γ-30–450, encoding the alpha and gamma isoforms, respectively (Fig. S2). The anti-γ antiserum is specific for Sdf-1γ, recognizing the same protein as the pan anti-Sdf-1 monoclonal MAB350 in cells over expressing Sdf1γ. Anti-γ showed no cross-reactivity with cellular proteins (Fig. S2). Thin sections of heart tissue were labelled to detect Sdf-1γ (anti-γ or MAB350), endothelial cells (anti-CD31) and myocardial cells (anti-troponin α). Both MAB350 (Fig. 1C) and anti-γ (Fig. 1D–F) labelled with comparable specificity a heart protein that was abundant in CD31+ endothelial cells lining the endocardium and in troponin α positive myocardial cells (Fig. 1C, E; arrowheads in insets). Single-cell resolution confocal immunofluorescence with both anti-Sdf-1 antibodies revealed a strong nuclear signal in troponin-α positive cells (Fig. 1C, E enlarged), and also in a fraction of endothelial cells, identified by localization and morphological criteria (Fig. 1C; asterisk in enlarged image), and all along the cardiac parenchyma (Fig. 1D). Furthermore, a punctate staining pattern typical of nucleolar localization (Fig. 1C–F) was observed in a significant proportion of cells with nuclear Sdf-1γ staining, suggesting targeting to the nucleolus.

### Nuclear targeting of Sdf-1γ maps to the specific carboxy-terminus

**Figure 2:**
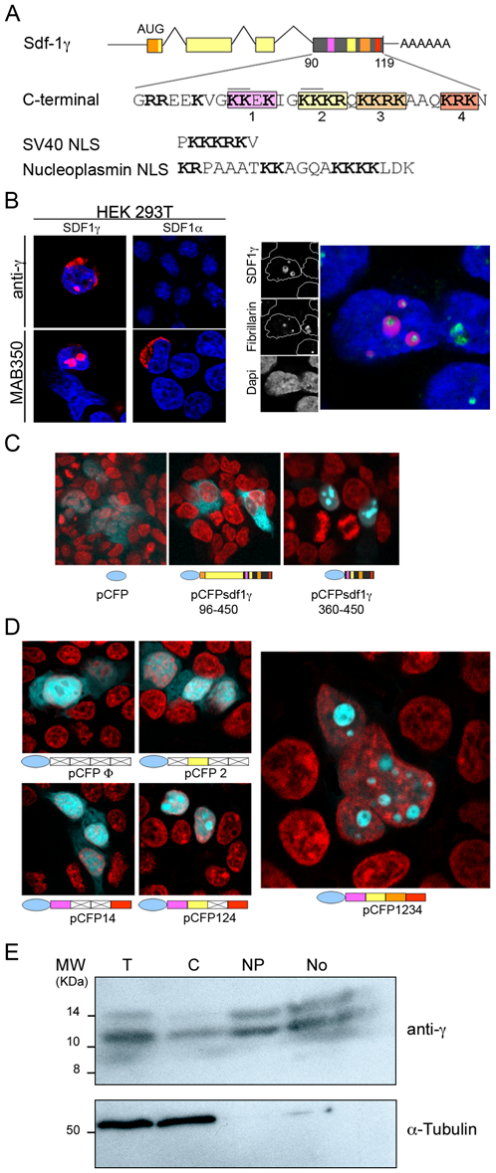
The Sdf-1γ carboxy-terminal region contains a nucleolar localization signal. (A) Schematic of the Sdf-1 locus showing the predicted Sdf-1γ exon structure, based on the annotated *Sdf-1*γ mRNA sequence from brain, and detail of the unique Sdf-1γ C-terminal exon 4. Basic amino-acid residues (Arg/Lys) are shown bold, and clusters of basic residues are boxed and numbered. SV40 and nucleoplasmin NLS sequences are shown for comparison. (B) Left panel. Confocal immunofluorescence of HEK293T cells transfected with plasmids pSdf1γ–30–450 (Sdf-1γ) and pSdf1α–30–450 (Sdf-1α). Subcellular localization of overexpressed Sdf-1 proteins (red) was detected with pan anti-Sdf-1 (MAB350) or specific anti-Sdf-1γ (anti-γ). Right panel. Co-labeling of the nucleolar protein fibrillarin and pSdf-1γ by confocal immunofluorescence. HEK293T cells were transfected with plasmid pSdf1γ–30–450 and immunostained for fibrillarin and Sdf-1γ. Individual and merged images of a representative field are shown on the left, and the inset regions are shown at high magnification on the right to show the localization of Sdf-1γ (green) and fibrillarin (red) in granular and fibrilar nucleololar regions, respectively. In both panels nuclei are stained with DAPI (blue). (C) Fluorescence images of HEK293T cells transfected with the indicated plasmids encoding cerulean fluorescent protein (CFP) fused to full-length Sdf-1γ (left) or the Sdf-1γ specific carboxy-terminus (center); The right panel shows results with unfused CFP. CFP fluorescence is shown blue, and nuclei are stained with TOPRO-3 (red). (D) Fluorescence images of HEK293T cells transfected with CFP fusions of wild-type or mutated versions of the Sdf-1γ C-terminal domain. Unmutated clusters of basic residues are colored as in A, and mutation (Lys/Arg→Ala) of the clusters is shown by the white/crossed boxes. Fluorescence signals are as in (C). Arrowheads indicate nucleoli. (E) Western blot of subcellular fractions of cell extracts. Cell equivalents were loaded on each lane. T, whole cell extract; C, cytoplasm; Np, nucleoplasm; No, nucleoli. Sdf-1γ was stained with anti-γ and developed with HRP-goat anti rabbit secondary antibody.

*In silico* sequence analysis of the SDF-1γ C-terminus identified a region enriched in basic amino acids (Lys and Arg) that shows high homology with classical NLS motifs of both the SV40 and bipartite nucleoplasm-like types (Fig. 2A) [19]. For ease of description, we have arranged the Lys and Arg residues into four clusters, numbered 1 through 4. To assess Sdf-1γ subcellular localization in detail, we ectopically expressed full-length Sdf-1γ in vitro. Plasmid pSdf1γ–30–450 contains a cDNA sequence corresponding to *Sdf-1γ* mRNA from brain according to annotated data (NM_001012477) (Fig. S1). Sdf-1γ was localized to the nuclei of almost 90% of transfected HEK293T cells, regardless of the antibody used (Fig. 2B). There was no evidence of Golgi accumulation of Sdf-1γ, in contrast to ectopically expressed Sdf-1α (Fig. 2B). To confirm nucleolar localization of Sdf-1γ, we transfected HEK293T cells with the pSdf1γ 96–450eGFP (encoding a C-terminal fusion of Sdf-1γ with eGFP; Fig. S1), and immunostained these cells for Sdf-1γ and fibrillarin, a nucleolar marker. Both proteins co-localized to the nucleolus, although Sdf-1γ mapped to the granular component, excluded from the fibrillar component labelled by fibrillarin.

To examine whether the nuclear localization signal for Sdf-1γ is located in the basic C-terminus, we fused the unique fourth exon encoding this region to the carboxyl end of cerulean fluorescent protein (CFP, pCFPSdf1γ–360–450). In HEK293T cells transfected with this plasmid the localization of CFP was unequivocally nuclear, with sub-localization to the nucleoli, and the same distribution was seen with CFP fused to full-length Sdf-1γ (pCFPSdf1γ−96−450) (Fig. 2C). The exon-4-encoded Sdf-1γ C-terminus is thus sufficient for nuclear localization, a finding confirmed by the fact that Sdf-1α and β, functionally equivalent to Sdf-1γ deletion mutants for this region, do not localize to the nucleus.

The contribution of each basic amino-acid cluster to Sdf-1γ nuclear localization was explored with mutant versions of pCFPSdf1γ–360–450 in which Lys and Arg residues were substituted by Ala, in accordance with natural mutation rates [23]. Construct pCFPΦ contains substitutions of all four clusters of basic residues; pCFP2 contains substitutions of clusters 1, 3 and 4; pCFP14 of clusters 2 and 3, and pCFP124 of cluster 3. Un-mutated pCFPSdf1γ–360–450 is represented here as pCFP1234. When expressed in HEK293T cells, un-fused CFP and CFPΦ distributed evenly throughout the cell, with no organelle-specific localization (Fig. 2D), discounting any significant role for passive diffusion in the redistribution of the low molecular weight Sdf-1γ. In contrast, CFP2 localized evenly throughout cell nuclei, indicating that cluster 2 (KKKR), which resembles the SV40-type NLS, is sufficient for nuclear redistribution. CFP14, maintaining integrity of clusters 1 (KKEK) and 4 (KRK), targeted the nucleus in the absence of cluster 2, but a proportion of transfected cells showed a cytoplasmic distribution. CFP124 was extensively sub-localized to nucleoli, similar to the distribution of the wild type sequence (CFP1234) (Fig. 2D). However, detailed observation of CFP1234 revealed localization to the granular component of nucleoli, in agreement with data obtained by co-labelling for fibrillarin (Fig. 2B right). This result indicates that exclusively nucleolar localization requires the presence of all four clusters, and based on this we can identify the putative NoLS as KKEKIGKKKRQKKRKAAQKRK.

These results were further confirmed by analysis of Sdf-1γ distribution in subcellular compartments (cytoplasm, nucleolus and nucleoplasm) in transfected HEK293T cells. Western blot of fractionated cultures revealed that the protein is expressed as two reactive species with apparent molecular weights of 12 and 14 KDa on polyacrylamide gels, representing larger products than Sdf-1α/β. Importantly, Sdf-1γ mostly accumulates in the nucleolar fraction and is almost completely excluded from the cytosol (Fig. 2E).

### Sdf-1γ is translated from a short mRNA expressed in cardiac tissue that skips the signal peptide encoded in the first exon

**Figure 3:**
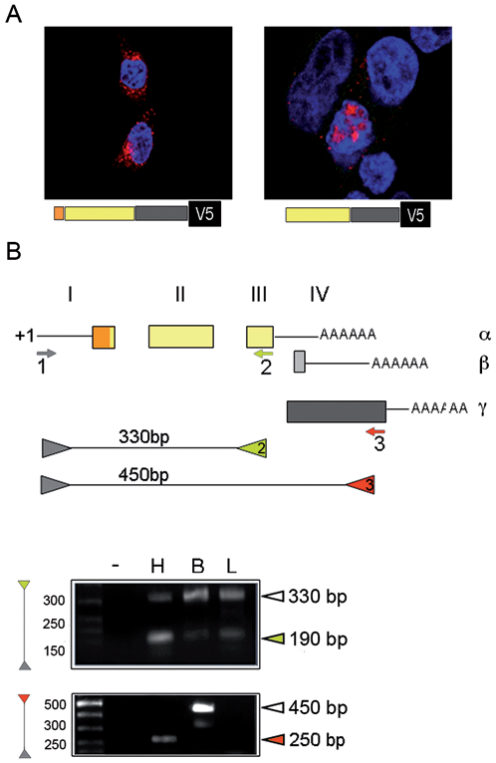
Expression of a specific Sdf-1γ mRNA in heart skips translation of the signal peptide. (A) Confocal immunofluorescence of C-terminally V5-tagged Sdf-1γ proteins. HEK293T cells were transfected with Sdf-1γ constructs including (left) or omitting (right) the N-terminal signal peptide sequence. Over expressed Sdf-1γ was detected with anti-V5 antibody (red). Nuclei were stained with DAPI (blue). (B) 5′ RACE was carried out on mRNA from adult mouse heart (H), brain (B) and liver (L), and cDNA products were amplified by nested PCR. The schematic represents the published annotated exon composition of *Sdf-1*α/β mRNA and *Sdf-1*γ, including exon 4. The predicted sizes of PCR products for mRNAs initiated at +1 are shown underneath. The positions of the RACE primers are shown by the small arrows 1–3, and the positions of corresponding PCR primers are shown by the coloured triangles. The gels show the actual products detected with the shared primer (green) and the γ-specific primer (red).

The published sequence for Sdf-1γ mRNA cloned from brain indicates that the translated protein should contain an N-terminal signal peptide, like Sdf-1α and β, together with the specific basic C-terminal end. To assess whether the exposed N-terminal signal peptide interferes with Sdf-1γ nucleolar localization, we ectopically expressed Sdf-1γ proteins labelled C-terminally with the cytoplasmic antigen V5. HEK293T cells transfected with pSdf1γ−30–450V5, encoding full-length Sdf-1γ, showed a diffuse immunofluorescence signal for V5, with accumulation in the perinuclear space but not in any structure resembling the Golgi apparatus (Fig. 3A). In contrast, an N-terminal deletion Sdf-1γ mutant lacking the signal peptide (encoded by pSdf1γ−151–450V5) predominantly localized to the nucleus in most transfected cells (Fig. 3A). These findings suggest that the opposing actions of the signal peptide and NoLS conflict when present in the same protein.

We next investigated whether *Sdf-1*γ transcription is subject to any process that counteracts the negative action of the signal peptide on nuclear accumulation. For this, we compared the *Sdf-1*γ mRNA species transcribed in adult brain and heart tissues which express high amounts of *Sdf-1*γ mRNA (Fig. 1A). RACE nested PCR assays were performed to identify transcription initiation sites, and the results compared with the *Sdf-1* species transcribed in liver. Use of common random RACE forward primers (grey arrowhead in Fig. 3B) in combination with specific reverse primers (coloured arrowheads) allowed us to specifically amplify *Sdf-1*γ mRNA (red) or a sequence common to all three isoforms (green). The major *Sdf-1*γ product amplified from brain was the predicted 450 bp sequence, but in heart a single band of about 250 bp was amplified. No *Sdf-1*γ band was amplified from liver, confirming lack of expression in this tissue. Sequencing revealed that the start site of the heart-specific *Sdf-1*γ transcript locates to nucleotide +145 downstream of the start site used for *Sdf-1*α/β (Fig. 3B). Interestingly, the cardiac *Sdf-1*γ transcripts lack the sequence encoding the signal peptide; this heart-specific transcript thus skips the AUG translation start codon used by Sdf-1α/β, present at the beginning of exon 1 (orange box in scheme in Fig. 3B). The *Sdf-1*γ transcript amplified in brain starts as predicted from the annotated +1 start site used for *Sdf-1*α/β.

Use of nested PCR with reverse primers directed to exon 3, common to all three isoforms (green arrowhead in Fig. 3B), identified a 330 bp product corresponding to transcription from the +1 start site in heart, brain and liver; however, the main product amplified in heart was a shorter band (190 bp), which sequencing showed to correspond to transcription of *Sdf-1*γ from the +145 start site. This finding is consistent with the expression of two proteins in different relative proportions revealed by Western blot (Fig. 2E). These data indicate that nuclear expression of Sdf-1γ in the heart is achieved by transcription of an mRNA with a shortened leader sequence (*slm*RNA) which omits the signal peptide.

### Translation of nuclear-targeted Sdf-1γ initiates from a non-canonical CUG initiation codon

**Figure 4:**
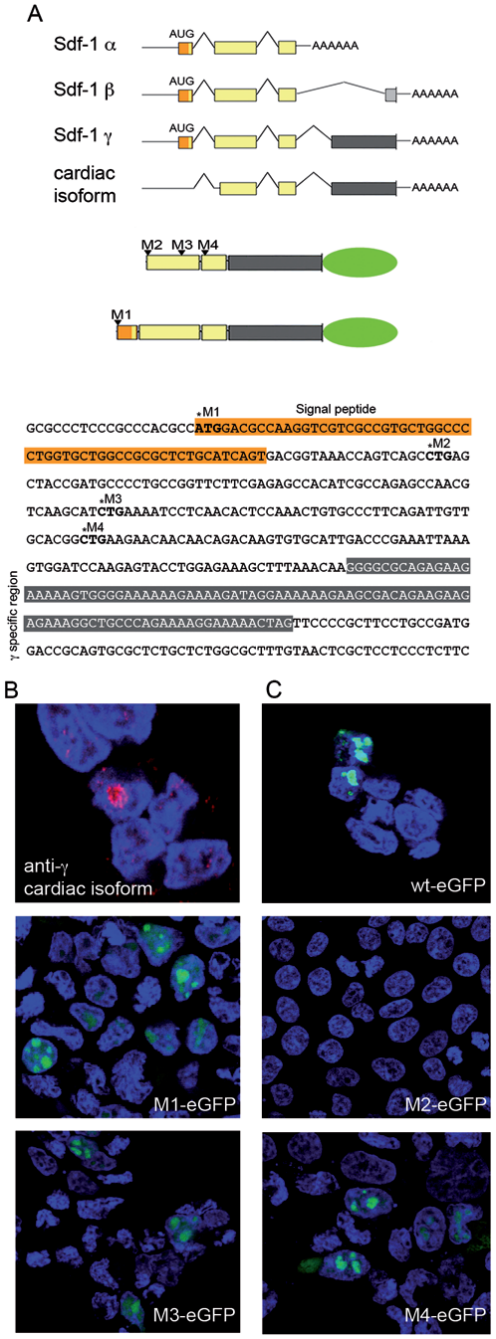
Expression of Sdf-γ uses a non-canonical CUG translation initiation codon. (A) Schematic representation of mRNA species for *Sdf-1*γ (*sl*mRNA) compared with *Sdf-1*α/β isoforms. The signal peptide is shown in orange and the specific 4^th^ exon of *Sdf-1*γ is in dark grey. The diagram below represents the SDF-1γ-eGFP tagged constructs containing CUG codons mutated (triangles). The nucleotide sequence corresponds to the cDNA for *Sdf-1*γ. The positions of the mutated non-canonical CUG-initiation codons are in bold, and named M2 to M4, and the mutation M1 of AUG common to Sdf-1α/β. (B) cDNA synthesized from *sl*mRNA obtained from cardiac tissue was cloned and the resulting plasmid (pcDNA-Sdf1γ-145-450) was transfected into HEK293T cells. Expression of Sdf-1γ was detected with specific anti-Sdf-1γ antibody (anti-γ). Nuclei were stained with DAPI (blue). (C) Plasmids encoding SDF-1γ-eGFP tagged constructs mutated in each of the CUG codons (M1 to M4) were transfected into HEK293T cells and the expression Sdf-1γ was monitored by eGFP fluorescence.

The absence of an AUG initiation codon from the *slm*RNA suggests an alternative mode of translation initiation for nuclear-targeted Sdf-1γ. To assess first whether the *slm*RNA transcript supports translation of Sdf-1γ, we transfected HEK293T cells with pcDNA-Sdf1γ-145–450, which contains the cDNA of *sl*RNA sequence (starting from +145, α/β numerals). Immunostaining with anti-γ and MAB350 antibodies confirmed high expression of the transfected protein and detected localization of the immunoreactive protein in the nucleolar compartment in the majority of transfected cells (Fig. 4B).

Non-canonical initiation codons have been described in a variety of proteins, and CUG seems to be the most common in metazoans [24]. Sequencing of a cDNA corresponding to *slm*RNA (Fig. 4A) indicated the presence of several in-frame CUG codons that are compatible with the synthesis of a 12 kDa protein (Fig. 2E). To determine which of the CUG codons is used we constructed a series of C-terminally eGFP-tagged expression plasmids derived from pcDNA-Sdf1γ-145–450, in each of which a single CUG codon is mutated to CUA, which does not function as an initiation codon [25] (Fig. 4A). As a control, pcDNASdf1γ-145–450, encoding the whole cDNA obtained from *sl*mRNA, was also assayed. When transfected into HEK293T cells, the mutations at position 232 (M3 in Fig. 4C) and 280 (M4) did not affect the expression of Sdf-1γ-eGFP (Fig. 4C), whereas the 169CUG mutation did (M2 in Fig. 4C). Thus CUG 169 is necessary for translation, suggesting that it is the non-canonical initiation codon used to translate Sdf-1γ from *sl*mRNA in the heart.

### Sdf-1γ nucleolar localization is unrelated to degradation

Nuclear localization is a feature of protein degradation via the nuclear ubiquitin-proteasome system (nUPS), and inhibition of this pathway increases accumulation of proteins targeted for degradation [26]. Sdf-1γ accumulation in nucleoli of transfected cells might therefore be linked to nUPS-mediated degradation, perhaps as a regulatory or quality control mechanism. To test this, we transfected HEK293T cells with pcDNASdf1γ-145–450, and treated them with MG132, an inhibitor of the proteasome ligase system. Nucleolar accumulation of Sdf-1γ was not increased by the treatment (Fig. S3), indicating that the Sdf-1γ turnover is not affected by proteasome activity and therefore that it localizes to the cell nucleoli through an active mechanism.

## Discussion

The findings presented here show that the major Sdf-1/Cxcl12 transcript (*sl*mRNA) encoding the γ isoform in cardiac tissue is translated from the non-canonical initiation codon CUG formed at the junction of exons 1 and 2. The chemokine is therefore expressed without a signal peptide, escaping the secretory pathway and allowing nucleolar localization mediated by the basic C-terminal domain. Sdf-1γ thus joins the expanding number of dual-function proteins, among which intrakines are the most prominent examples. Chemokines form part of the complex cytokine network, and there is growing evidence that some cytokines of the IL-1 and FGF families have intracellular actions; for example IL-1α precursor [27], IL-33 [28], ESkine Ccl27 [29] and parathyroid hormone [30]. However, all reported functions of chemokines are mediated through binding to their membrane-bound, or occasionally soluble, receptors, and no pathway has been described for intracellular localization of chemokines. These findings are thus the first indication that chemokines can exert functions through pathways unrelated direct intercellular communication.

Nucleolar localization suggests novel, as yet undefined, autocrine functions for Sdf-1γ in cardiac cells. Given its structural and functional distinction from the other protein products of the *Sdf-1* gene, including Sdf-1γ expressed in brain, we propose that the non-secreted form of Sdf-1γ expressed in cardiac tissue be renamed *C*ardiac *D*erived *F*actor 1 (Cdf-1). The human and rat orthologs are highly homologous, and a similar subcellular distribution can be envisaged in the adult heart, where Sdf-1γ is also expressed [13], [14].

Our results show high expression of *Sdf-1*γ mRNA in the heart after birth and in adulthood. Previously reported *in situ* hybridization analysis of *Sdf-1*, using probes common to all isoforms, detected expression in the developing mouse heart [22] and in adulthood [18]. Similarly, *Sdf-1* knockout mice give no information on isoform-specific functions, since gene targeting of *Sdf-1* affects all three isoforms [8]; nonetheless, embryos deficient for *Sdf-1* show defects in septum formation in the developing heart, and based on our qRT-PCR data it seems likely that loss of *Sdf-1*γ expression is involved in this defect. Recently Franco et al [31] have studied by imunohistochemistry and non quantitative RT-PCR the expression pattern of *Sdf-1* during mouse development with special attention to Sdf-1γ. The results of these authors are substantially different to our quantitative data, particularly regarding expression in adult and neonatal heart of Sdf-1γ and Sdf-1β in the liver. A detailed observation of their data shows defects in actin controls beside the technique employed by those authors is not accepted as quantitative. Importantly, our data support the idea that in cardiac tissue the signal obtained by Franco et al in the heart with K15 antibody correspond to isoform –α and –β as the region recognized by the antibody K15C is encode in the N-terminal end of the protein ([31] and references therein) that is skipped during expression of Sdf-1γ in the heart, as we describe in this paper.

The *sl*mRNA appears to be the most abundant *Sdf-1*γ transcript expressed in the adult mouse heart, whereas brain expresses the longer transcript, which includes the signal peptide sequence. Although some minor expression of the *sl*mRNA form might occur in brain, our results suggest that Sdf-1γ proteins in heart and brain are likely to be synthesized as different forms, possibly contributing to their different fates and functions in each tissue. Further experiments will be needed to determine the relevance and functional implications of such differences.

In our experiments we examined Sdf-1γ protein expression by Western blot of transfected cloned cDNA derived from *Sdf-1*γ mRNA from brain. This mRNA species contains the exon encoding the signal peptide, and gives rise to two specific bands of approximately 14 and 12 KDa on polyacrylamide gels (Fig. 2E). These apparent sizes are consistent with the translation of one protein from the ATG codon (119 amino acids) and one from the CUG codon starting at position 169 (93 amino acids) described in this study. These data suggest either that ribosomes are able to initiate translation from two different codons in the same mRNA or that there are two mRNAs, the *sl*mRNA and the larger species containing the ATG codon. Further studies on the transcription and translation of this gene in the brain are required to explore the diversity and specificities of the regulation of the *Sdf-1*γ gene. Expression in cardiac tissue of the short Sdf-1γ with no signal peptide is consistent with the data obtained by Segret et al [18], showing that in vivo in rat cardiac tissue, Sdf-1γ is an intracellular 12 kDa protein.

### Conflicts on the journey toward the cell nucleus

Sdf-1γ contains an NLS/NoLS that we have mapped to the exon-4-encoded C-terminal domain. The presence of a secretory signal motif and a NLS in the same protein creates potential for conflictive or complex regulation of protein fate. Signal peptides direct proteins toward the endoplasmic reticulum, and several studies have deciphered the pathways by which certain dual-function cytokines and growth factors can re-enter the cell complexed to internalized target receptors and are transported to intracellular sites such as the nucleus (for a review, see [31]). In other cases, protein distribution is determined by competition between the signals for secretion and nuclear localization [32]. However, no receptor-mediated internalization mechanism has been reported for proteins such as Sdf-1γ that bind to seven transmembrane domain G-protein coupled receptors [31]. Instead, our data indicate that expression of Sdf-1γ in the heart follows a novel nuclear localization strategy involving alternate mRNA processing and translation initiation.

The viability of *slm*RNA is confirmed by translation of Sdf-1γ from cDNA cloned from heart tissue, and the mutational analysis demonstrates that translation of this transcript is initiated from codon CUG at position 26 (α/β aa numbering). CUG is the most common alternative initiation codon in metazoans [25], and more than a dozen mammalian genes have been reported to produce isoforms from non-AUG codons [24]. The CUG codon itself is unlikely to confer cardiac specificity, since translation from this codon is widely observed in lymphoid [33], neuronal [34] and endothelial [35] cells. The CUG initiation codon for cardiac expression of Sdf-1γ is downstream of the first in-frame AUG codon, a feature shared only with the alternative translation initiation of osteopontin (Opn) [33]. However, in the case of Sdf-1γ CUG usage appears to be determined by a transcriptional control mechanism, rather than mRNA secondary structure as in the case of Opn.

It is not clear by what mechanism the short *Sdf-1*γ mRNA is specifically synthesized in cardiac cells. There may be a specific mechanism for transcription initiation that omits the signal peptide sequence, or this sequence might be eliminated by the action of an RNA exonuclease. Without the signal peptide sequence, the *slm*RNA has a first exon shorter than any reported [36], which raises questions about how such a short oligonucleotide sequence can be spliced to the second exon. Whatever the mechanism, it will be of interest to determine how this process is triggered exclusively in the heart, and whether similar processes occur in other tissues and with other cytokines.

Nucleolar localization signals are thought to interact with structural nucleolar proteins or RNAs, but no consensus sequence requirement has been identified for such signals beyond a grouping of basic residues [37]. Our findings suggest that proper nucleolar localization of Sdf-1γ requires the whole set of basic residues present in the C-terminal domain, arranged as four clusters. We therefore propose the sequence KKEKIGKKKRQKKRKAAQKRK as a novel NoLS present in Sdf-1γ.

The nucleolus is the most prominent subnuclear structure, and is involved in ribosome subunit assembly. The accumulated data suggest that the nucleolus contains a dynamic proteome of more than 400 proteins and associates transcription regulatory networks via several mechanisms [21], including degradation pathways mediated by the ubiquitin–proteasome system operating in the nucleus (nUPS) and nucleolar-cytoplasm or nucleolar-nucleoplasm protein transit. Based on this last activity, the nucleolus can be regarded as a reservoir of regulatory proteins, acting as a sequestering compartment for regulatory complexes, or regulating the exposure of proteins to proteolysis during developmental decision-making (Hand 1) [38], hypoxia sensing responses (VHL) [39], cell death and proliferation (AKT, p53, ARF, MDM2, c-myc) and mitosis (Cdc14). Consistently, nucleolar targeting is associated with regulatory mechanisms involved in cellular commitment, and, intriguingly, during cardiac development [40]. Our results show that nucleolar accumulation of Sdf-1γ is unaffected by inhibition of the nUPS, suggesting that nucleolar accumulation of Sdf-1γ is unrelated to protein quality-control [26], [41]. It therefore appears that Sdf-1γ in the heart is localized to the nucleolus to exert its biological function. Our preliminary data indicate that overexpression of Sdf-1γ seems not to be required for the induction of apoptosis or to promote any alteration of the cell cycle.

### Concluding remarks

Organismal complexity relies partly on the capacity of genomes to generate protein diversity. Such diversity is commonly achieved through alternative gene expression pathways (alternative transcriptional initiation, mRNA splicing and translation initiation), allowing related proteins to acquire domains that confer novel activities [42]. Since the first description of intracellular forms of growth factors for FGF family members and their receptors [reviewed in [43]], several growth factors and cytokines have been shown to be expressed both as secreted and intracellular forms (nuclear or cytoplasmic). By including or deleting key domains from their structures, proteins can be targeted to particular cell compartments and excluded from others. Such diversity must strike a balance between protein stability, compatible signalling and multifunctionality.

We have shown that a product of the chemokine *Sdf-1* gene is specifically directed to the nucleolus through a combination of transcriptional and translational mechanisms. The specific nucleolar expression of *Sdf-1*γ in the heart suggests important functions in this organ and provides the first example of a nucleolar directed chemokine. These new properties of this isoform will lead to novel insights into the functions encoded by the *Sdf-1* gene.

## Materials and Methods

### Plasmid Construction

Plasmids were generated from PCR products with the pGEM-T Easy system (Promega Biotech Ibérica SL, Alcobendas, Spain). All primers used are listed in Table S1. After sequencing, inserts were excised by restriction digestion and subcloned in the HindIII site of the destiny vector pmCerulean-C1 (Clontech, Saint-Germain-en-Laye, France) to drive expression of fluorescent fusion proteins. For overexpression of proteins by either transfection or transduction, inserts were subcloned in the XbaI site of pLV series lentiviral vectors, derived from the HIV-based pRRsyn18 vector. All vectors were verified by sequencing with an ABIPrism 3000 sequencer. The final constructs are shown in Fig. S1. Plasmids harboring mutated versions of the basic-residue clusters of the C-terminal region of Sdf-1γ were synthesised by DNA2.0 (Basel, Switzerland) and subcloned into HindIII and XbaI restriction sites of pmCerulean-C1.

### Mammalian Cell Culture and Transfection

The human embryonic kidney cell line HEK293T and the Chinese hamster ovary (CHO) cell line were cultured under standard conditions in DMEM (Cambrex) supplemented with 1% Glutamax (Invitrogen, Prat del Llobregat, Barcelona, Spain), 10 mg/ml antibiotics (penicillin streptomycin) and 10% foetal bovine serum (BioWhitaker). Cells were transfected with endotoxin-free DNA (Qiagen, Las Matas, Madrid, Spain) and transfections were carried out in 6-well plates unless otherwise stated. HEK293T cells were transfected by the calcium-phosphate method, and CHO cells were transfected using Lipofectamine 2000 (Invitrogen, Prat del Llobregat, Barcelona, Spain). For proteasome inhibition experiments, HEK293T cells were grown in 6-well plates, and incubated for 8 h in the presence of 10 mM carbobenzoxy-L leucyl-L-leucyl-L-leucinal (MG132, Calbiochem, Nottingham, UK) diluted in DMSO (Sigma Aldrich, Alcobendas, Spain). Untreated and DMSO treated cells were cultured in parallel as controls.

### Mouse samples

Mice of the inbred strain C57BL/6 were purchased from the Jackson Laboratory (Bar Harbor, Maine) and bred in our animal facility under standard conditions. Female mice (8–12 weeks old) were checked daily for pregnancy, and were anesthetised at the indicated times and embryos removed aseptically. Embryonic heart, bone marrow, liver, brain, yolk sac and AGM were extracted, and tissues from 5–8 dams per time point were pooled and used for RNA extraction. Adult tissues were extracted from euthanized animals and individual samples were collected and used for total RNA isolation.

Animals were housed in accordance with Spanish law for the protection of animals used for experimental and other scientific purposes, Real Decreto 1201/2005, which enacts EU Directive 86/609/EEC. Housing and husbandry conditions conform to the Council of Europe Commission Recommendation of 18 June 2007 on guidelines for the accommodation and care of animals used for experimental and other scientific purposes. The CNIC Ethical Review Committee approved all animal procedures.

### Antibodies

A rabbit polyclonal antiserum (anti-γ) was raised against the peptide KVGKKEKIGKKKRQ, mapping to the specific C-terminal region of Sdf-1γ (see Fig. S2). The N-terminal cysteine enables direct conjugation of the peptide to the protein carrier and is not present in the native sequence. Peptide synthesis, coupling, immunization, ELISA titration and affinity purification were done by BioGenes GmbH (Berlin, Germany).

Commercial antibodies used were mouse monoclonal anti pan-Sdf-1 clone 79018.111 (R&D System, Abingdon, UK), rabbit anti-fibrillarin - nucleolar marker (ab5821) (AbCam, Cambridge, UK), goat anti mouse Troponin T (AbCam,), rabbit anti-mouse CD31 (Sigma Aldrich) and mouse anti V5 antibody (Molecular Probes, Invitrogen). Secondary antibodies used were Alexa-488-conjugated goat anti-rabbit, Alexa-488-conjugated goat anti-mouse, Alexa-633-conjugated goat anti-rabbit and Alexa-633-conjugated goat anti-mouse (Molecular Probes, Invitrogen).

### Confocal immunofluorescence and immunohistochemistry

For subcellular localization of antigens by double-label indirect immunofluorescence (IF), cells were seeded onto glass coverslips coated with poly-L-Lysine (Sigma), transfected or transduced. 24 to 48 hours later, cells were washed twice with PBS and fixed (5 min) in cold 4% paraformaldehyde, permeabilized with 0.5% TritonX-100/PBS and blocked with 4% normal goat serum. Thereafter samples were incubated (45 min, 37°C) with anti-γ (1/100 dilution), MAB350 (1/200) or anti-fibrillarin antibody (1/xxx). Primary antibodies were diluted in PBS supplemented with 5% goat serum.

The secondary antibodies Alexa-633-conjugated goat anti-rabbit or Alexa-633-conjugated goat anti-mouse (Molecular Probes) were used at 1/500. Finally, samples were dyed with ToPro3 or DAPI (Invitrogen) to stain DNA, air dried and mounted in Gelatin (Sigma Aldrich). Samples were examined with a Leica SP4 confocal laser scanning microscope (Leica Microsystems Holdings, Wetzlar Germany) fitted with two lasers giving excitation at 488 nm and 633 nm (for secondary antibodies) and 633 nm (ToPro3) or 405 nm (DAPI). Data were collected sequentially at a resolution of 1024×1024 pixels from 0.5–1.0 µm thick optical slices.

Immunohistochemistry was performed on OCT-preserved mouse heart samples. Thin (5 µm) cryosections were fixed in 4% paraformaldehyde. Antigens were retrieved by incubating samples in citrate buffer for 30 min at 85°C. Sections were blocked in 5% goat serum (Sigma Aldrich) and double labeled with anti-γ serum (1/100) or MAB350 (1/1000) together with anti troponin T (AbCam: 1/200) or anti CD31 (Sigma Aldrich: 1/500). Antibodies were diluted in PBS and incubations conducted overnight in a humidified chamber at 4°C. Anti-mouse or anti-rabbit secondary antibodies (Molecular Probes, Invitrogen) were diluted 1/500 in PBS and incubations were conducted under similar conditions. Sections were counterstained with DAPI and mounted with Gelatin (Sigma Aldrich). Slides were examined with a Leica SP5 confocal microscope (Leica Microsystems Holdings).

### RNA Extraction and qRT-PCR

Total RNA was extracted from tissues and cell cultures using Trizol (Sigma Aldrich) according to the manufacturer's procedure, followed by a treatment with RNase-free DNase (Roche Applied Science, Sant Cugat del Vallès, Barcelona, Spain). Aseptically removed organs were homogenized in extraction buffer with an Ultra Turrax T8 mechanical homogenizer (Janke & Kunkel, Staufen, Germany), and RNA was purified from cleared lysates.

cDNA was synthesized from 500 ng total RNA using a Superscript III First Strand cDNA synthesis kit (Invitrogen). Amounts of specific mRNAs in samples were quantified by qRT-PCR using an ABIPrism 7900 HT Detection System (Applied Biosystems, Foster City, CA, USA) and SYBER green detection. PCR was performed in 96-well microtest plates (Applied Biosystems) with 0.5 units of Taq Polymerase (Applied Biosystems) per well and 35-40 cycles. In all experiments, mRNA amounts were normalized to the total amount of cDNA by using amplification signals for 18S and GAPDH. Each sample was determined in triplicate, and at least three independent samples of each tissue or cell line were analyzed. The efficiency of each primer pair was measured using the Real-Time PCR MINER software (http://miner.ewindup.info/miner/). Primer sequences and PCR conditions are listed in Table S1.

### RACE

Two pairs of primers were used for first and nested amplifications. 5′RACE was conducted with the SMART^TM^ RACE cDNA amplification kit (Clontech), following the manufacturer's instructions. Briefly, a first cDNA synthesis was performed with oligo dT primers supplied by manufacturer, and dC residues were introduced by the reverse transcriptase when it reached the mRNA end. These cDNAs were then annealed to SMART II A oligonucleotides, which serve as a template for subsequent PCR reactions in combination with specific reverse primers. The primer sequences used for this assay are detailed in Table S1. Specific primers targeted exon 4 and common primers targeted exon 3. The products of nested PCR were cloned into pGEM-T easy vector and sequence analyzed.

### Western blot

Proteins were extracted following standard procedures in the presence of Complete Protease Inhibitor Cocktail Tablets (Roche Applied Science). Western blots were carried out by standard methods on proteins transferred to PVDF using TransFi (Invitrogen). Membranes were probed for Sdf-1 with MAB350 (1/1000) or anti-γ serum (1/200) in PBS/0.1% Tween-20 (PBS-Tween). Secondary antibodies were HRP-conjugated goat anti-mouse IgG or goat anti-rabbit IgG (Santa Cruz Biotechnology, Inc., Heidelberg, Germany), and blots were developed with ECL (GE Healthcare, Alcobendas, Spain) as previously described [44].

### Cell fractionation

Cytoplasm, nucleoplasm and nucleoli were purified as previously described [45]. Briefly, 80×10^6^ exponentially growing HEK293T cells were scraped into cold TNMK buffer (50 mM Tris-HCl, 130 mM NaCl, 5 mM KCl, 8 mM MgCl_2_, pH 7.2), and a sample representing whole-cell extract was retained. Cells were then pelleted and resuspended in RSB-5 hypotonic buffer (10 mM Tris-HCl, 10 mM NaCl, 5 mM Mg acetate, pH 7.4), allowed to swell on ice for 30 min, and lysed by the addition of NP40 to a final concentration of 0.3%. After Dounce homogenization, the nuclear fraction was recovered by low speed centrifugation and the cytoplasmic supernatant was retained. Nuclei were further purified by centrifugation through 0.88M sucrose and resuspended in 0.34M sucrose plus 0.5 mM magnesium acetate (sonication buffer), and were sonicated to release the nucleoli. These were purified by two centrifugation cycles through 0.88M sucrose: the supernatant of the first cycle is the nucleoplasm fraction and the pellet of the second cycle corresponds to the nucleolar fraction. Pellets were adjusted to represent cell equivalent fractions of the starting material.

## Supporting Information

Table S1 List of Primers used
(0.05 MB DOC)

Figure S1 (A) Genetic organization of mouse Cxcl12. Schematic representation of annotated mRNA species for Sdf-1γ and compared that of Sdf-1α/β isoforms. Common exons to the three isoforms are depicted by yellow boxes, the signal peptide in orange and the specific 4th exon of Sdf-1β or γ in green or respectively. Numerals under the exons indicate the residue numbers starting in the Met of α and β isoforms. Numbers in the upper scale refers to nt starting in +1 of Sdf-1α/β mRNAs. (B) Schematic representation of the plasmids used along this investigation containing the depicted mouse Cxcl12 cDNAs. Color are as in (A) and the specific 4th exon of Sdf-1γ is shown in black with coloured bars representing groups of basic (Lys and Arg) residues (see Fig. 2A).
(2.89 MB TIF)

Figure S2 (A) The sequence of the peptide used to raise the anti-γ antibody is indicated by a yellow box below the enlarged specific 4th exon of Sdf-1γ. (B) Western blot of HEK293T total cell extracts obtained fron transfected cultures with the indicated plasmids expressing either Sdf-1α/β or Sdf-1γ and revealed with affinity purified anti-γ serum (left) or the commercial pan SDF-1 MAB350 antibody and were developed with HRT-goat antirabbit or HRT-goat antimouse, respectively.
(1.17 MB TIF)

Figure S3 Inhibition of proteosome protein-degradation with MG132 has no effect on nucleolar accumulation of Sdf-1γ. HEk293T cells were transfected with pcDNA-Sdf1γ-134-450 (Fig. 4B) and after 24 h the proteosome inhibitor MG132 was added to the next 6 h. Asfterward cells were fixed and stained for Sdf-1γ with anti-γ. Cells were treated with the olvent DMSO and sowed as MOCK. Nuclei are staine in blue with DAPI.
(6.89 MB TIF)
