# Supplementary material for: A Chemokine Targets the Nucleus: Cxcl12-Gamma Isoform Localizes to the Nucleolus in Adult Mouse Heart
Source: PLoS One. 2009 Oct 27;4(10):e7570. doi: 10.1371/journal.pone.0007570 (PMC2762742; doi:10.1371/journal.pone.0007570)
Supplement: Table S1 — List of Primers used (0.05 MB DOC) [file pone.0007570.s001.doc]

Supplementary TABLE 1. List of Primers used

| Primer name | Application | Sequence |
| --- | --- | --- |
| Cxcl12 Fw | Real Time PCR | CCAACCTGTGCCCTTCAGATTG |
| Cxcl12 α Rv | Real Time PCR | CATATGCTATGGCGGAGTGTC |
| Cxcl12 β Rv | Real Time PCR | CTTCAGCCCTGCTCAGGAGC |
| Cxcl12 γ Rv | Real Time PCR | ACTGCGGTCCATCGGCAGG |
| 18s Fw | Real Time PCR | AAACGGCTACCACATCCAAG |
| 18s Rv | Real Time PCR | CCTCCAATGGATCCTCGTTA |
| GAPDH Fw | Real Time PCR | CAAGGTCATCCATGACAACT |
| GAPDH Rv | Real Time PCR | TCACGCCACAGCTTTCCAGA |
| Cxcl12 common | RACE | TTTCAGATGCTTGACGTTGGCTCTGGC |
| Cxcl12 γ | RACE | GTTTTTCCTTTTCTGGGCAGCCTTTCTC |
| Nested Cxcl12 common | RACE | CAGCCGTGCAACAATCTGAAGGG |
| Nested Cxcl12 γ | RACE | TTCTGGGCAGCCTTTCTCTTCTTCTGTC |
| Universal Primer (Clontech) | RACE | CTAATACGACTCTACTATAGGGC |
| NUP (Clontech) | RACE | AAGCAGTGGTATCAACGCAGAGT |
| Cxcl12 Fw | Cloning | CGTCTAGAGCTGTCCAGCTCTG |
| Cxcl12 Rv | Cloning | CGTCTAGAGGAGGAGCGAGTTAC |
