# Supplementary figures and images for: A Chemokine Targets the Nucleus: Cxcl12-Gamma Isoform Localizes to the Nucleolus in Adult Mouse Heart
Source: PLoS One. 2009 Oct 27;4(10):e7570. doi: 10.1371/journal.pone.0007570 (PMC2762742; doi:10.1371/journal.pone.0007570)

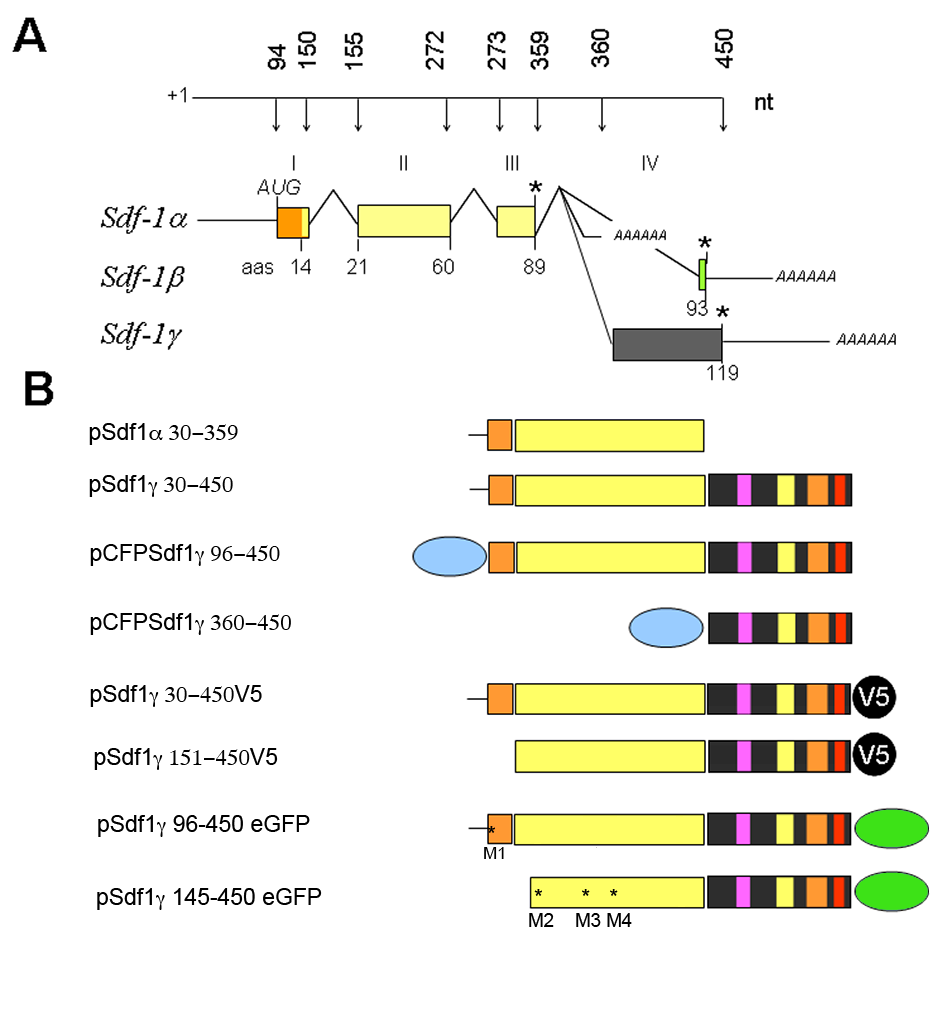

Supplement: Figure S1 — (A) Genetic organization of mouse Cxcl12. Schematic representation of annotated mRNA species for Sdf-1γ and compared that of Sdf-1α/β isoforms. Common exons to the three isoforms are depicted by yellow boxes, the signal peptide in orange and the specific 4th exon of Sdf-1β or γ in green or respectively. Numerals under the exons indicate the residue numbers starting in the Met of α and β isoforms. Numbers in the upper scale refers to nt starting in +1 of Sdf-1α/β mRNAs. (B) Schematic representation of the plasmids used along this investigation containing the depicted mouse Cxcl12 cDNAs. Color are as in (A) and the specific 4th exon of Sdf-1γ is shown in black with coloured bars representing groups of basic (Lys and Arg) residues (see Fig. 2A). (2.89 MB TIF) [file pone.0007570.s002.tif]

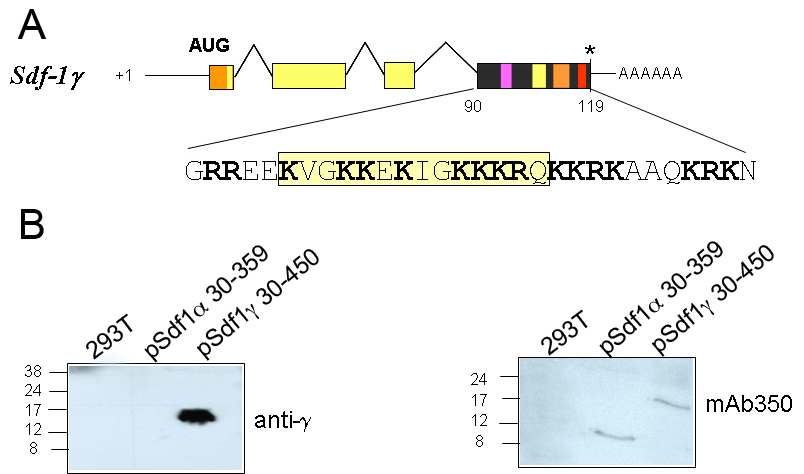

Supplement: Figure S2 — (A) The sequence of the peptide used to raise the anti-γ antibody is indicated by a yellow box below the enlarged specific 4th exon of Sdf-1γ. (B) Western blot of HEK293T total cell extracts obtained fron transfected cultures with the indicated plasmids expressing either Sdf-1α/β or Sdf-1γ and revealed with affinity purified anti-γ serum (left) or the commercial pan SDF-1 MAB350 antibody and were developed with HRT-goat antirabbit or HRT-goat antimouse, respectively. (1.17 MB TIF) [file pone.0007570.s003.tif]

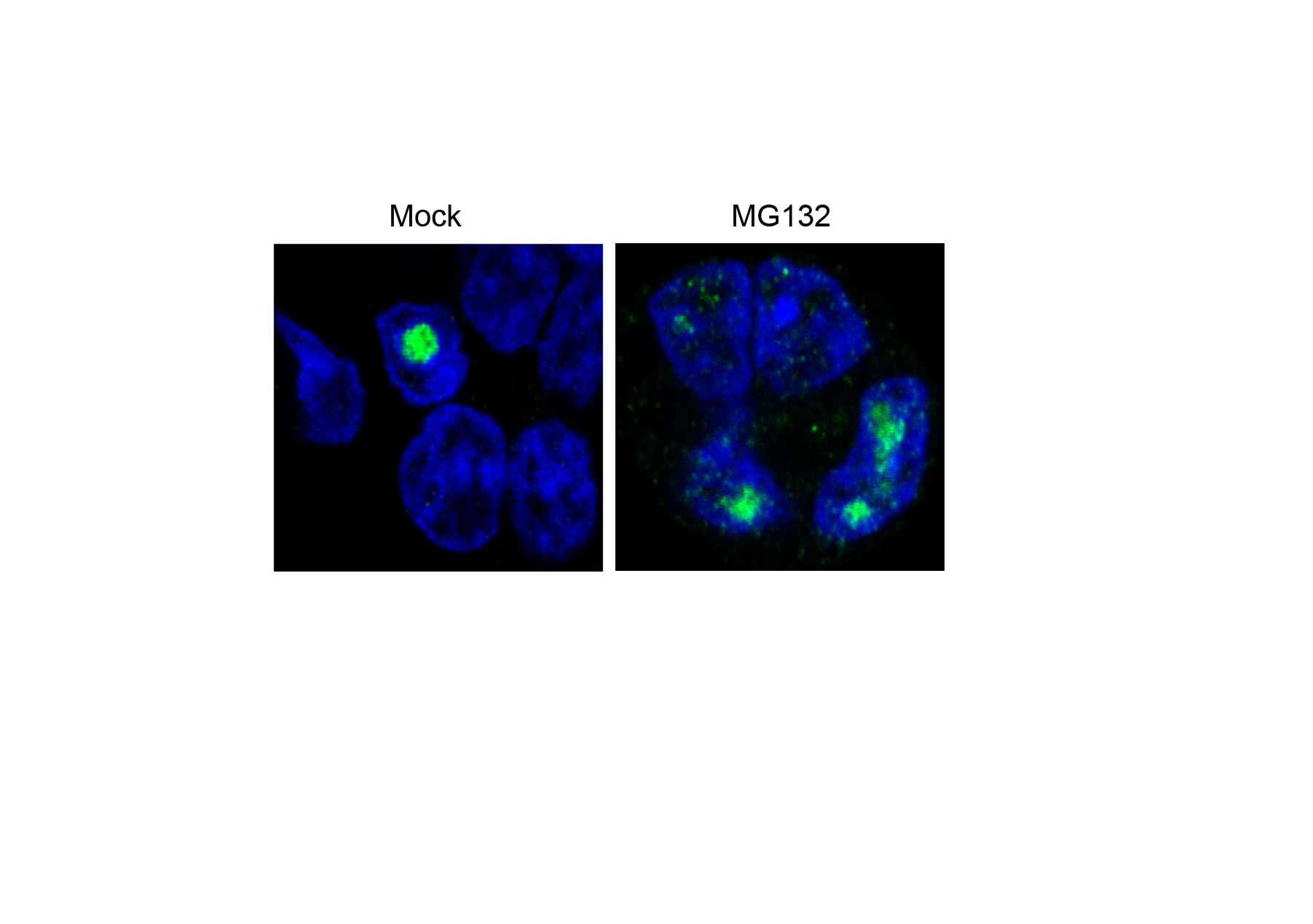

Supplement: Figure S3 — Inhibition of proteosome protein-degradation with MG132 has no effect on nucleolar accumulation of Sdf-1γ. HEk293T cells were transfected with pcDNA-Sdf1γ-134-450 (Fig. 4B) and after 24 h the proteosome inhibitor MG132 was added to the next 6 h. Asfterward cells were fixed and stained for Sdf-1γ with anti-γ. Cells were treated with the olvent DMSO and sowed as MOCK. Nuclei are staine in blue with DAPI. (6.89 MB TIF) [file pone.0007570.s004.tif]
